# Cerebrospinal fluid flow modulates brain health

**DOI:** 10.1172/JCI197202

**Published:** 2025-09-02

**Authors:** Lauren M. Hablitz, Maiken Nedergaard

**Affiliations:** 1 Center for Translational Neuromedicine, University of Rochester Medical Center, Rochester, New York, USA.; 2 Center for Basic and Translational Neuroscience, Faculty of Health and Medical Sciences, University of Copenhagen, Copenhagen, Denmark.

## Abstract

Cerebrospinal fluid dynamics play an important role in maintaining brain health and clearing metabolic waste from the brain. In this issue of the *JCI*, Gursky et al. investigate how CSF distribution is affected when its primary efflux pathway — the deep cervical lymph nodes — is disrupted by cauterization. This timely study reveals compensatory fluid drainage routes from the skull, age-dependent adaptations in CSF homeostasis, and the emergence of neuroinflammation when an efflux pathway is occluded. The findings underscore the need to better understand the physiological mechanisms governing CSF clearance, how these pathways evolve with aging, and whether CSF influx and efflux exhibit region-specific dynamics shaped by neuroanatomy. Additionally, the study raises important questions about whether peripheral injury can influence central nervous system states. A more complete understanding of CSF flow regulation may offer new perspectives on the origins of neuropathology.

## Cerebrospinal fluid pathways in the central nervous system

Cerebrospinal fluid (CSF) plays a vital role in maintaining brain health, yet fundamental questions remain about how CSF moves within the central nervous system and exits the skull. In the past decade, renewed interest has emerged around CSF production, subarachnoid space flow dynamics, and its critical role in metabolic waste clearance from the brain parenchyma.

CSF first appears in the ventricles, where it is produced primarily by the choroid plexus and potentially supplemented by fluid influx across the blood-brain barrier (BBB) (1). The choroid plexus is a specialized epithelial structure that facilitates ionic exchange, concentrates signaling molecules and neuromodulators into the CSF, and plays a role in immune surveillance of the blood-CSF interface (2). Once secreted, CSF circulates through the ventricular system and enters the subarachnoid space, which is enclosed by multiple meningeal and dural layers that function as both physical barriers and immune-regulatory interfaces (3, 4).

From the subarachnoid space, CSF can flow caudally into the spinal cord, drain directly into cervical lymph nodes, or enter the glymphatic system, a network of perivascular conduits defined by astrocyte endfeet and aquaporin-4 (AQP4) channels that direct CSF influx into brain tissue and facilitate interstitial solute clearance (5). From there, brain interstitial fluid drains into meningeal and cervical lymphatic vessels, along the carotid artery, and along cranial nerves, ultimately being dumped back into the venous system (6).

The relative drainage of CSF among the multiple efflux paths is dynamically regulated by arousal state (7), circadian timing (8), anesthesia (9, 10), and body posture (11). Disruption of this fluid balance — such as by lymphatic blockage — can exacerbate neuroinflammation, as seen in preclinical models of traumatic brain injury (12). A deeper understanding of how CSF selects specific drainage pathways under different physiological conditions may be central to unraveling the etiology of diseases where glymphatic dysfunction is implicated.

**Figure 1:**
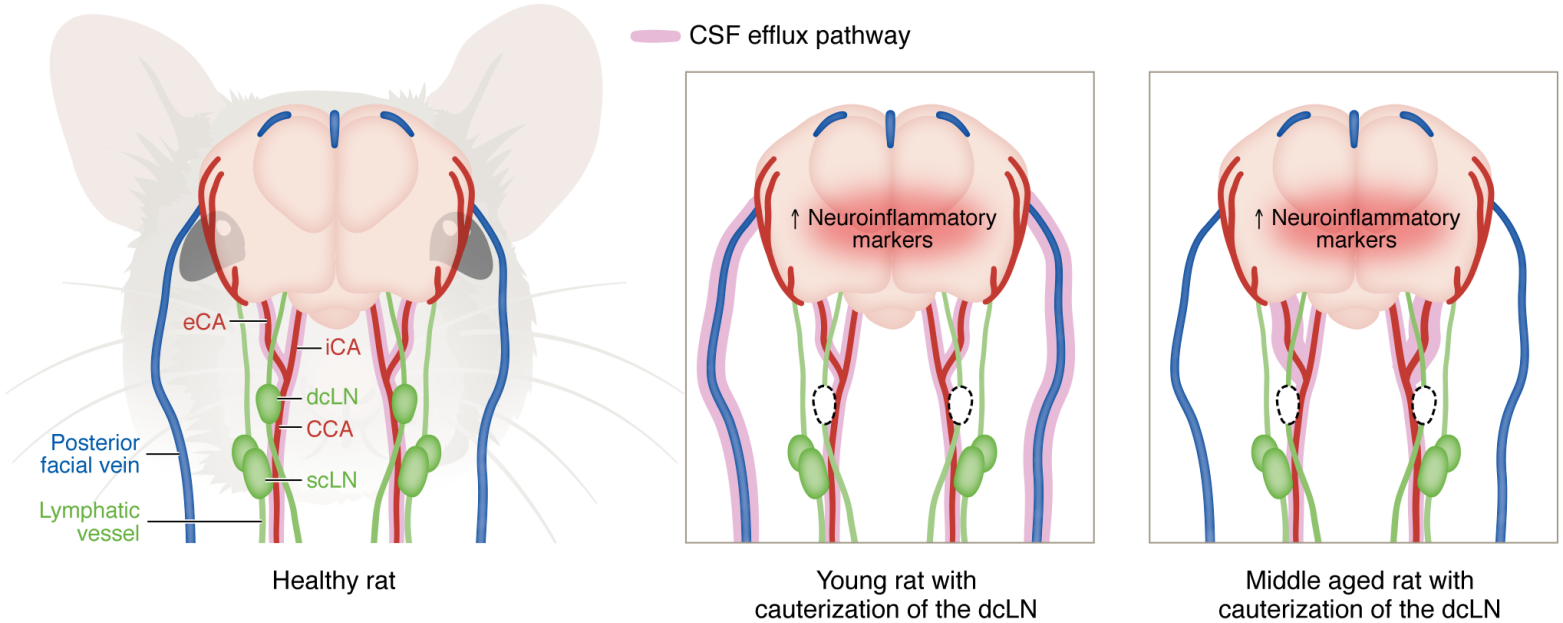
Rerouting of CSF drainage after cauterization of the deep cervical lymph nodes. In healthy rats (left), cerebrospinal fluid (CSF) efflux pathways generally follow a carotid artery pathway (red) from the internal and external carotid arteries (iCA and eCA, respectively) toward the common carotid artery (CCA), accompanied by drainage by the lymphatic system (green), with accumulation in the deep and superficial cervical lymph nodes (dcLN and scLN, respectively). Following cauterization of the dcLN (dotted outlines), young rats (middle) exhibited a rerouting of efflux (highlighted in pink) towardsthe posterior facial veins (blue), in addition to existing carotid artery pathways. In middle-aged rats (right), efflux increased along the eCA, while no change was observed along the cervical veins. Omics analysis indicated elevated neuroinflammatory markers in both young and middle-aged rats following dcLN cauterization (depicted as darker brain color). Efflux of CSF contrast agent is depicted in pink.

Gursky et al. (13) address a fundamental question to the field of brain fluid dynamics: What happens to CSF distribution when access to the deep cervical lymph nodes is obstructed? Using dynamic contrast-enhanced MRI (DCE-MRI) in rats, the authors demonstrated that after cauterization of the deep cervical lymph nodes, efflux is rerouted through the cervical veins and the carotid vasculature, with concurrent alterations in glymphatic influx and clearance (Figure 1). Importantly, this disruption is associated with elevated neuroinflammatory markers in the brain. Below, we highlight several key implications of this study.

## Aging and the glymphatic/lymphatic systems

Rodent studies have shown that glymphatic function declines in mid- to late life, coinciding with changes in astrocytic AQP4 localization and increased gliosis (14). Reduced vascular pulsatility and diminished cervical lymphatic contractility further impair CSF clearance with age (15). In mice, glymphatic decline can begin as early as 10 months of age (16).

Gursky et al. (13) compared CSF tracer distribution in young (3-month-old) and middle aged (10-month-old) rats following intracisternal injection. In sham-operated rats, efflux was observed along the superficial cervical lymphatics and the carotid arteries. After deep cervical lymph node cauterization, young rats exhibited rerouting of CSF to the posterior facial veins, while middle-aged rats showed increased drainage via the external carotid artery, with minimal involvement of cervical veins. These age-dependent shifts in efflux routes suggest that the lymphatic drainage pathways underwent compensatory remodeling, which may have implications for the progression of neurodegeneration and the efficacy of intrathecal drug delivery. Future research should evaluate whether such compensations exist in humans, and whether factors like body posture modulate these routes.

## Regional vulnerability and subregion-specific glymphatic dynamics

Most studies of glymphatic influx and clearance have focused on the cerebral cortex due to its large periarterial spaces and accessibility for imaging, as well as technical limitations related to imaging deep into the parenchyma. However, other brain regions such as the brainstem and cerebellum remain understudied, despite their distinct vascular and astrocytic architecture. Regional differences in vascular tortuosity, astrocyte density, or perivascular space morphology could result in “glymphatically vulnerable” areas prone to protein aggregation and neuropathology.

Gursky et al. (13) applied kinetic modelling of CSF tracer dynamics and observed that deep cervical lymph node cauterization reduced tracer influx but paradoxically accelerated clearance in the brainstem and cerebellum. These results were accompanied by increased neuroinflammatory signatures across the brain.

While reduced tracer influx and accumulation of neuroinflammatory markers is expected following obstruction of CSF efflux, accelerated clearance is counterintuitive. This surprising observation may reflect nonglymphatic shunting of CSF from the injection site at the cisterna magna, the largest section of the subarachnoid space. Given the injection site’s anatomical proximity of the brainstem and cerebellum to the injection site, tracer washout may have occurred through local CSF pathways independent of perivascular transport. The possibility of tracer washout raises an important methodological consideration: tracer injection location may influence regional clearance estimates and interpretation of glymphatic function. An alternative — though highly speculative — hypothesis is that the observed neuroinflammation and altered tracer kinetics do not directly indicate glymphatic dysfunction, but instead reflect a secondary response to shear stress caused by excessive CSF flow.

## Sampling lymphatic CSF composition to monitor neurological disease

CSF is increasingly used as a biomarker-rich fluid to monitor neurological disease progression. In Alzheimer’s disease, for example, the Aβ42/Aβ40 ratio in CSF is a key diagnostic tool (17). In the present study, Gursky et al. reported that dcLN ablation altered CSF proteomic, metabolomic, and lipidomic profiles, consistent with elevated neuroinflammation (13).

These findings highlight a critical but often overlooked point: CSF composition is shaped by its route, state of brain activity, and residence time. For example, suppression of glymphatic efflux eliminates the presence biomarkers of traumatic brain injury in blood (18). Ventricular CSF likely contains different molecular content than CSF from the cisterna magna or spinal canal, due to proximity to the brain parenchyma and interaction time with interstitial solutes. Moreover, CSF collection methods are invasive. The study prompts an intriguing question: could lymph fluid, sampled from cervical lymphatics, offer a less invasive yet informative surrogate for brain-derived pathology in both animal models and humans?

## Conclusions

Gursky et al. (13) provide compelling evidence that aging and disruption of primary CSF efflux routes reshape the fluid drainage pathway from the skull and enhance neuroinflammatory responses. Their findings support the concept of a flexible, state-dependent clearance network, shaped by age, anatomy, and lymphatic integrity.

To close, we pose a speculative but thought-provoking question: If CSF follows a conserved anatomical route from the ventricles to peripheral lymph nodes, could disorders of the peripheral lymphatic system (i.e. beyond the cervical nodes), such as infections or injury to distal organs, retrogradely signal into the brain and trigger inflammation or pathology? Exploring this possibility may reveal new dimensions of brain-body immune communication and ultimately inform novel strategies for the diagnosis and treatment of neurodegenerative diseases.

## Version 1 09/02/2025

Electronic publication
